# Characterisation of the circulating acellular proteome of healthy sheep using LC-MS/MS-based proteomics analysis of serum

**DOI:** 10.1186/s12953-017-0119-z

**Published:** 2017-06-10

**Authors:** Saul Chemonges, Rajesh Gupta, Paul C. Mills, Steven R. Kopp, Pawel Sadowski

**Affiliations:** 1 School of Veterinary Science, The University of Queensland, Gatton, Australia; 2 Proteomics and Small Molecule Mass Spectrometry, Central Analytical Research Facility, Queensland University of Technology, Brisbane, Australia

**Keywords:** Sheep serum, Ovine circulating acellular proteome, nanoLC-nanoESI-MS/MS, Gene ontology, Protein pathway analysis, Sheep serum proteomics, Proteogenomics data

## Abstract

**Background:**

Unlike humans, there is currently no publicly available reference mass spectrometry-based circulating acellular proteome data for sheep, limiting the analysis and interpretation of a range of physiological changes and disease states. The objective of this study was to develop a robust and comprehensive method to characterise the circulating acellular proteome in ovine serum.

**Methods:**

Serum samples from healthy sheep were subjected to shotgun proteomic analysis using nano liquid chromatography nano electrospray ionisation tandem mass spectrometry (nanoLC-nanoESI-MS/MS) on a quadrupole time-of-flight instrument (TripleTOF® 5600+, SCIEX). Proteins were identified using ProteinPilot™ (SCIEX) and Mascot (Matrix Science) software based on a minimum of two unmodified highly scoring unique peptides per protein at a false discovery rate (FDR) of 1% software by searching a subset of the Universal Protein Resource Knowledgebase (UniProtKB) database (http://www.uniprot.org). PeptideShaker (CompOmics, VIB-UGent) searches were used to validate protein identifications from ProteinPilot™ and Mascot.

**Results:**

ProteinPilot™ and Mascot identified 245 and 379 protein groups (IDs), respectively, and PeptideShaker validated 133 protein IDs from the entire dataset. Since Mascot software is considered the industry standard and identified the most proteins, these were analysed using the Protein ANalysis THrough Evolutionary Relationships (PANTHER) classification tool revealing the association of 349 genes with 127 protein pathway hits. These data are available via ProteomeXchange with identifier PXD004989.

**Conclusions:**

These results demonstrated for the first time the feasibility of characterising the ovine circulating acellular proteome using nanoLC-nanoESI-MS/MS. This peptide spectral data contributes to a protein library that can be used to identify a wide range of proteins in ovine serum.

**Electronic supplementary material:**

The online version of this article (doi:10.1186/s12953-017-0119-z) contains supplementary material, which is available to authorized users.

## Background

There is currently no publicly available reference mass spectrometry-based circulating acellular proteome data for sheep. However, the well-defined serum proteome of humans permits analysis and interpretation of a range of physiological changes and disease states [1, 2]. To date, the serum proteome of sheep is largely extrapolated from cattle, which can be inaccurate despite a 97% similarity in protein coding sequences [3] and different promoters driving the expression of specific proteins [4]. Characterisation of the serum proteome of sheep would therefore be useful to quantify disease in this species.

Sheep are a major production species, providing meat and wool, plus are used in a range of biotechnological and translational studies [5–9]. Despite this, relatively little is known about the responses of sheep to a range of physiological and pathological events, including the effects of breed differences in these responses. There is therefore a need to comprehensively characterise the proteins in ovine serum for better quantitative assessment of disease and any alternations in physiology and pathology. Blood is relatively easily collected from sheep [10–14], but comparatively only a small number of proteins have been identified, limiting the capacity to assess disease [10, 15]. One problem to date is that protein sample preparation in published studies on sheep have been inadequate and have generally ignored the full conventions for reporting identified proteins from samples [16, 17]. Consequently, data are lacking on optimised sample preparation approaches for shotgun proteomics workflows using more than one protein sequence search engine to explore the circulating acellular proteome of sheep. For example, the number of proteins identified by single laboratories using gel fractionation followed by MS from human plasma has been in the region of nearly 300 protein identifications (IDs) [18]. In 2005, liquid chromatography tandem mass chromatography (LC-MS/MS) data from multiple sample preparation techniques and protein sequence search engines for the Human Plasma Proteome Project (HPPP) from 18 laboratories worldwide collectively identified 3,020 plasma proteins based on a minimum of 2-high-scoring peptides [19, 20]. This number of protein IDs from HPPP studies was subsequently revised to 889 [19, 21]. A study that used high performance liquid chromatography (RP-HPLC) and LC-ESI-MS/MS to analyse and define the human baseline plasma proteome identified 200 proteins [22]. More recently, protein expression profiles of human plasma proteins using one-dimensional sodium dodecyl sulfate polyacrylamide gel electrophoresis (1D SDS–PAGE) coupled with nanoLC–ESI–MS/MS in a single laboratory identified 253 proteins after desalting of the peptides [23]. A similar approach to that used in the preceding study was considered attractive to be used in exploring the circulating acellular proteome of sheep.

The present study used nano liquid chromatography nano electrospray ionisation tandem mass spectrometry (nanoLC-nanoESI-MS/MS) to analyse peptides derived from healthy sheep serum samples following 1D SDS–PAGE and in-solution digestion.

## Methods

### Overview of methods

This study used universal protein extraction techniques detailed hereinafter to comprehensively define the serum proteome of healthy sheep. Because of the genome of sheep being incompletely sequenced or annotated, proteins were identified by matching tryptic peptides against a composite protein sequence database of sheep, goat and ox using ProteinPilot™ Software (SCIEX) in the first instance in order to capture homologous sequences. The inclusion of protein sequences from related species is a helpful strategy when exploring and establishing foundation proteogenomics data to identify known or novel genes of the non-model study subject — in this case sheep [24–30]. Mascot [31] (Matrix Science) search was subsequently conducted using a sheep-only protein sequence database to identify high-scoring proteins and PeptideShaker [32] (CompOmics, VIB-UGent) to verify protein identifications from the primary search data.

### Animal care, sample collection, storage and preparation

Serum samples of healthy adult female Merino sheep (*n* = 6) with ear tag identification numbers 473, 413, 463, 471, 476 and 478 belonging to an experimental colony at Queensland University of Technology (QUT) and the Australian Red Cross Blood Service (ARCBS) were obtained for the development and optimisation of a comprehensive proteomic approach for interrogating the circulating acellular proteome. The sheep were reared according to established standard operating procedures, described elsewhere [33]. Sample aliquots of 500 μL were stored in 1.5 mL Eppendorf tubes at -80 °C at the ARCBS, Brisbane. The samples were transferred to the wet laboratory at the Molecular Genetics Research Facility (MGRF) within Central Analytical Research Facility (CARF), QUT for processing. The processed samples were analysed by nanoLC-nanoESI-MS/MS at the Proteomics and Small Molecule Mass Spectrometry laboratory at CARF, QUT.

### Sample preparation for protein analysis

Frozen sheep serum samples were thawed on ice and then centrifuged at 13,000 *g* at 4 °C for 20 min. The sediment and top layer comprising mainly of lipids and suds were discarded, retaining the supernatant. The protein concentration in the supernatant was determined with bicinchoninic acid (BCA) protein assay kit (BCA Protein Assay Kit, Pierce™) according to the manufacturer’s instructions using a spectrophotometer (NanoDrop 2000, Thermo Scientific). The supernatant was then either directly analysed or concentrated by acetone precipitation of proteins. In some experiments, a protease inhibitor cocktail tablet (Roche) was added into the sample after thawing, according to the manufacturer’s instructions.

### Acetone precipitation of proteins

Proteins in serum were precipitated by adding 4 × (v:v %) of cold (-20 °C) acetone and then incubated at -20 °C for 16 h, prior to centrifugation at 4,000 *g* for 2 min. The supernatant was discarded. The pellet was washed with cold acetone and the suspension was centrifuged at 4,000 *g* for 5 min at 4 °C. The supernatant was discarded and this procedure was repeated one more time. The pellet was then dissolved in freshly prepared 8 M urea in 25 mM ammonium bicarbonate (NH_4_HCO_3_) (Sigma-Aldrich) buffer. The mixture was centrifuged at 4,000 *g* for 5 min at 4 °C, the supernatant was kept and the insoluble sediment was discarded. The protein concentration of the supernatant was determined using the BCA method [34].

### 1D SDS-PAGE

The universal 1D SDS-PAGE procedure used to fractionate proteins was based on its established description [35] and subsequent refinements [36–39]. The detailed description is provided in Additional file 1.

The gels were stained with Coomassie brilliant blue (EZ-Run™, Protein Gel Staining Solution, Fisher Scientific) according to the manufacturer’s instructions and then photographed using a handheld camera (5.7-inch Quad HD Super AMOLED®, Samsung; or New 8-megapixel iSight camera with 1.5 μ pixels with Optical image stabilisation, iPhone 6, Apple Inc.).

Gel bands from entire single lanes were excised into 12 approximately equal portions into a clean 1.5 mL Eppendorf tube and de-stained using 50% acetonitrile (ACN) (Optima®, Fisher Scientific) in 25 mM NH_4_HCO_3_ accompanied by agitation at 750 rpm for 20 min at RT. This procedure was repeated and alternated with washing the gel bands with 25 mM NH_4_HCO_3_ buffer. Once de-stained, final washing of the gel bands was performed using LC-MS grade water followed by incubation for 20 min at RT. The water was discarded and the gel bands were cut into approximately 1 mm^3^ pieces using a 10 uL pipette tip. Gel bands were dehydrated by adding 100% ACN and agitating at 750 rpm for 10 min at RT prior to drying in a vacuum centrifuge (SpeedVac Concentrator Christ® cat. No. RVC 2-33 IR), for 10 min.

In-gel proteins were reduced in order to break disulphide bonds and alkylated to prevent the bonds re-forming as originally described elsewhere [40]. Briefly, freshly prepared 10 mM DTT (Sigma-Aldrich) in 25 mM NH_4_HCO_3_ buffer was added sufficiently to cover the vacuum dried gel pieces and agitated at 750 rpm for 45 min at 56 °C. Twice the amount of DTT as of freshly prepared 55 mM iodoacetamide (IAM) (Sigma-Aldrich) in 25 mM NH_4_HCO_3_ buffer was added to the sample and agitated for 30 min at RT in the dark. The reagents were washed off with 25 mM NH_4_HCO_3_ buffer with agitation for 5 min at RT, before centrifuging briefly and discarding the supernatant. Gel bands were then dehydrated using 100% ACN and agitated at 1400 rpm for 10 min at RT. The entire supernatant was discarded prior to drying the gel pieces in a vacuum centrifuge as above for 20 min.

Vacuum-dried gel pieces were incubated on ice for 5 min before adding 0.005 μg/μL solution of freshly prepared ice-cold working solution of trypsin (Trypsin Gold, Mass Spectrometry Grade, Promega) in 50 mM NH_4_HCO_3_ buffer enough to cover the dry gel pieces [41] and left incubating for a further 30 min until the entire enzyme solution had entered the gel pieces. Gel pieces were then covered in 50 mM NH_4_HCO_3_ buffer and left to incubate for 16 h at 37 °C on an agitator at 300 rpm. Digestion was stopped by adding 100 μL of 5% formic acid (FA) (Sigma-Aldrich). Peptide extraction was performed by agitating the gel pieces at 1,000 rpm for 15 min at RT. The peptide-containing supernatant was collected into a clean 0.5 ml low binding Eppendorf tube. Gel pieces were further washed by adding 5% FA in 50% ACN and agitating at 1,000 rpm for 15 min, before collecting the supernatant. Gel bands were further extracted by adding 100% ACN and agitation at 1,000 rpm for 15 min at RT. The entire supernatant was collected and then completely vacuum-dried prior to reconstitution in 10 μL of 0.1% trifluoroacetic acid (TFA) (Sigma-Aldrich) in 2% ACN followed by desalting of peptides.

### In-solution digestion of proteins

The method adapted here was based on the one established by Villén and Gygi [42]. Briefly, a known quantity of serum or plasma protein sample was thawed on ice at 4 °C after which freshly prepared 20 mM DTT (equal v:v% of sample) was added, vortexed and briefly centrifuged. The mixture was diluted fivefold with 25 mM NH_4_HCO_3_ buffer (v:v% of sample) to dilute down urea concentration below 1 M, followed by adding an equivalent (v:v% of sample) of aqueous 70 mM CaCl_2_. Trypsin was then added at enzyme to substrate (protein concentration of sample) ratio of 1:50. The contents were incubated for 16 h at 37 °C and then cooled to RT. Digestion was stopped by adding 50 μL of 10% TFA before vacuum concentrating the contents to dryness. The dried peptides were reconstituted in aqueous 0.1% TFA in 2%ACN, and followed by desalting of peptides.

### Desalting of tryptic peptide digests

It is often necessary to remove salts and particulate matter including excess trypsin from peptide digests prior to analysis to prevent blockage of nanoLC columns and also to reduce noise artefacts of MS spectra [43–45]. Desalting of tryptic peptide digests was optimised and performed using either octadecyl carbon chain (C_18_) pipette tips (ZipTip® Pipette Tips, Millipore, or Pierce C_18_ Tips, Thermo Fisher Scientific) depending on the filter capacity according to manufacturer’s instructions. Briefly for the C_18_ tips, the desalting pipette tip was conditioned using a solution of 50% ACN/0.05% trifluoroacetic acid (TFA) in LC-MS grade water (Optima®, Fisher Scientific) and then equilibrated with 2% ACN/ 0.1% TFA in LC-MS water. After carefully and gently pipetting the entire sample up and down for at least 10 times, the membrane was washed with 2% ACN/0.1% TFA in LC-MS water. The peptides were eluted using 70% ACN/0.1% TFA in LC-MS water, vacuum dried and reconstituted in 10 uL of 2% ACN/0.1% FA in LC-MS water and transferred into a polypropylene autosampler vial for nanoLC-nanoESI-MS/MS analysis.

## nanoLC-nanoESI-MS/MS

### Chromatography

Peptide spectral data from approximately 400 ng – 1 μg of injected tryptic peptides per sample were generated using nanoLC-nanoESI-MS/MS on a TripleTOF® 5600+ System (SCIEX) instrument. Peptides were separated by performing reversed-phase chromatography using an Eksigent ekspert™ nanoLC 400 System directly coupled to the MS/MS instrument. The LC platform was setup in a trap and elute configuration with a 10 mm × 0.3 mm trap cartridge packed with ChromXP C18CL 5 μm 120 Å material and a 150 mm × 75 μm analytical column packed with ChromXP C18 3 μm 120 Å (Eksigent Technologies, Dublin, CA). The mobile phase solvents were composed of mobile phase A: water/0.1% FA; mobile phase B: ACN/0.1% FA; and mobile phase C: water/2% ACN/0.1% FA. Trapping was performed in mobile phase C for 5 min at 5 uL/min followed by an elute configuration across a 90 min gradient using two mobile phases A and B. To minimise retention time drift, the analytical column was maintained at 40 °C.

### Data dependent acquisition (DDA)

The DDA mode of the instrument was set to obtain high resolution (30,000) TOF-MS scans over a mass range of 350–1350 *m/z*, followed by up to 40 (top 40) high sensitivity MS/MS scans of the most abundant peptide ions per cycle. The selection criteria for the peptide ions included intensity greater than 150 cps and charge state of 2–5. The dynamic exclusion duration was set at 12 s to account for the difference in chromatographic peak width matching to the peaks in the chromatogram. Each survey (TOF-MS) scan lasted 0.25 s and the product ion (MS/MS) scan lasted 0.05 s resulting in a total cycle time of 2.3 s. The ions were fragmented in the collision cell using rolling collision energy, and CES was set to 5. The collected peptide ion fragmentation spectra were stored in .wiff format (SCIEX).

### Data processing

#### Primary protein sequence database search for protein identification

The acquired MS/MS data from the instrument were extracted and annotated with amino acid sequences from a custom built database using the Paragon™ Algorithm: 5.0.0.0, 4767 [46] (ProteinPilot™ Software 5.0, Revision Number: 4769, SCIEX, USA.). The custom composite database (62,025 sequences; 29,099,284 residues) used in Paragon™, with added common contaminants was assembled in FASTA format downloaded on 29^th^ July, 2015 from a repository of non-redundant and predicted protein sequences of *Ovis aries, Bos taurus* and *Capra hircus* sourced from UniProtKB (Universal Protein Resource Knowledgebase - http://www.uniprot.org/). Another sheep (*Ovis aries*) only custom database (27,393 sequences, 13,114,569 residues) with added contaminants from The common Repository of Adventitious Proteins, cRAP (http://www.thegpm.org/crap/) was assembled in FASTA format (26 Jul, 2016) from UniProtKB was used for sheep protein validation. For ProteinPilot™ searches, the following settings were selected: Sample type: Identification; Cys Alkylation: Iodoacetamide; Digestion: Trypsin; Instrument: TripleTOF 5600+; Special Factors: Urea denaturation; Species: None; Search effort: Thorough ID; ID Focus: Amino acid substitution; Results Quality: Detected protein threshold [Unused ProtScore (Conf)] ≥ 0.05 with false discovery rate (FDR) selected. Annotations were only retrieved from UniProt during composite searches. The automatically generated Excel spreadsheet (Microsoft® Excel 2010, Microsoft Corporation) report in ProteinPilot™ output was manually inspected for FDR cut-off protein yields and then meticulously curated to filter out contaminants, protein identifications with 0 (zero) unused confidence scores, proteins with reversed (nonsense) sequences and redundant protein IDs. Only proteins identified at FDR ≤1% with ≥ 2 peptides were considered for protein lists and for visual comparative analysis in the first instance and further downstream analysis.

The .group file data in ProteinPilot™ were exported as calibrated Mascot generic format (.mgf) and mzIdentML (.mzid) format files. The .mgfs were further reformatted by an mgf repair tool (SCIEX) to recalibrate .mgf files so that they can be parsed to recognise the boundaries between original files and avoid collisions in spectrum identifiers, prior to loading via a Daemon application to Mascot search engine (Matrix Science, London, UK; version 2.5.1) [31]. Mascot was set up to search the same custom database that was used in ProteinPilot™ with the following search parameters: type of search: MS/MS ion search; enzyme: trypsin; fixed modifications: Carbamidomethyl (C); variable modifications: deamidated (NQ), oxidation (M); mass values: monoisotopic; protein mass: unrestricted; peptide mass tolerance: ± 10 ppm; fragment mass tolerance: ± 0.01 Da; max missed cleavages: 1; instrument type: ESI-QUAD-TOF, and the auto-decoy search option was selected. Protein identifications were made at a significance threshold of *p* < 0.05 or target decoy of 1% FDR. Peak list and identification data from the search were exported in a .dat format for further processing. Protein lists were exported in csv format for immediate data evaluation and curation to remove contaminants in Excel spreadsheet. Only proteins identified with 2 or more peptides were included for further evaluation.

#### Secondary protein sequence database search for protein identification and validation

The .mgf, .dat and .mzIdentML (from ProteinPilot™) files were also loaded for protein identification and validation using PeptideShaker [32]. Peak lists obtained from MS/MS spectra were identified using Mascot [31]. Protein identification was conducted against a concatenated target/decoy [47] version of the *Ovis arie*s (27,284; 99.5%) complement of the UniProtKB, 27,411 (target) sequences. The decoy sequences were created by reversing the target sequences in SearchGUI. The identification settings were as follows: Trypsin with a maximum of 1 missed cleavages; 10.0 ppm as MS1 and 0.5 Da as MS2 tolerances; fixed modifications: carbamidomethylation of C (+57.021464 Da), variable modifications: deamidation of N (+0.984016 Da), deamidation of Q (+0.984016 Da), oxidation of M (+15.994915 Da), pyrolidone from E (--18.010565 Da) and pyrolidone from Q (--17.026549 Da), fixed modifications during refinement procedure: carbamidomethylation of C (+57.021464 Da). All algorithm-pecific settings are listed in the Certificate of Analysis available in the data files.

Peptides and proteins were inferred from the spectrum identification results using PeptideShaker version 1.13.0 [32]. Peptide Spectrum Matches (PSMs), peptides and proteins were validated at a 1.0% False Discovery Rate (FDR) estimated using the decoy hit distribution. All validation thresholds are listed in the Certificate of Analysis available in the data files. Post-translational modification localisations were scored using the D-score [48] and the phosphoRS score [49] with a threshold of 95.0 as implemented in the compomics-utilities package [50]. Protein identification reports were exported in .xlsx format for evaluation and curation in Excel spreadsheet. Only proteins identified with 2 or more validated peptides were included for further evaluation.

Protein lists were presented in spreadsheet and charts were made (Microsoft® Excel™ 2010, Microsoft Corporation). Data were visualised using BioVenn Software [51], where appropriate.

The mass spectrometry data along with the identification results were deposited to ProteomeXchange Consortium [52] via the proteomics identifications (PRIDE) partner repository [53] with the dataset identifiers PXD004989 and 10.6019/PXD004989 with the following data access details: Reviewer account details: Username: reviewer99399@ebi.ac.uk; Password: QBFFTGzl

### Analytical samples, experimental layout and data collection

In order to characterise the serum proteome of sheep, two universal sample preparation strategies for shotgun proteome analysis [54] were employed in three paired sets of experiments (first, second and third), using in-gel and in-solution protein digestion of serum samples. This was followed by peptide analysis by nanoLC-nanoESI-MS/MS using the method described above.

### 1D SDS-PAGE of normal sheep serum workflow

As a pilot study, an acetone precipitated serum sample obtained from one sheep (Sheep ID 473) was processed and subjected to 1D SDS-PAGE to ascertain the feasibility of obtaining protein identification data as a basis for constructing a peptide spectral library in future (First in-gel digestion). In order to determine the optimum amount of serum protein to load, 2, 10 and 22 μg of protein were run in separate wells of the same gel. To determine the amount of protein that needed to be loaded on a gel for protein bands to be visualised after using EZ-Run protein stain, 250, 500 and 2500 fmol of bovine serum albumin (BSA) protein were loaded in separate wells of another gel and run.

In order to increase the protein coverage, a fraction of acetone precipitated serum sample from Sheep ID 473 was subjected to 1D SDS-PAGE in two gels run concurrently (second in-gel digestion). One gel was loaded with 50 μg and 100 μg of protein in adjacent lanes and the second gel was also loaded with 50 μg, 100 μg and 50 μg in adjacent lanes.

In order to determine the effect of the quantity of protein loaded, acetone precipitation and a protease inhibitor on protein coverage, pooled serum samples from six healthy sheep (Sheep IDs 413, 463, 471,473, 476 and 478) were processed and subjected to 1D SDS-PAGE in three gels (third in-gel digestion). The samples utilised consisted of crude protein (200 μg and 100 μg) on one gel and then 100 μg of acetone precipitated serum protein with or without a protease inhibitor (Roche) and 100 μg of crude serum in a second gel. A third gel was loaded and run identically as the second gel.

### In-solution digestion of sheep serum workflow

As a pilot study, 10 μg of acetone precipitated serum sample obtained from one sheep was subjected to in-solution digestion to ascertain the feasibility of obtaining protein identification data as a basis for protein quantitation in future (first in-solution digestion). In order to determine the effect of using unfractionated sample on protein coverage, a fraction of 20 μg of crude serum sample from the sheep used in the first in-gel digestion was subjected to in-solution digestion and analysed (second in-solution digestion). A third experiment utilised 100 μg of pooled crude serum samples from all six sheep (Sheep IDs 473, 413, 463, 471, 476 and 478) for in-solution digestion in order to determine the effect of using a higher quantity of protein substrate on protein coverage (third in-solution digestion).

## Results

**Fig. 1:**
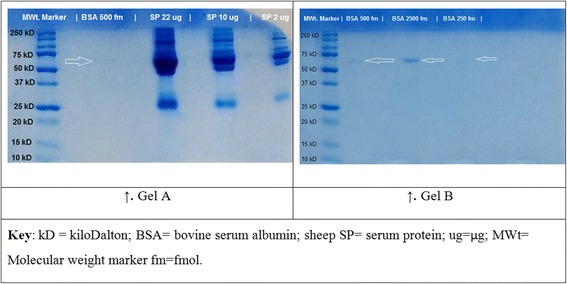
Coomassie-stained 1D SDS-PAGE gels used in first in-gel digestion. Fractions of acetone precipitated serum protein from a healthy sheep were loaded alongside bovine serum albumin (BSA) in Gel A. Gel A suffered a handling artefact to the *top right corner* of the gel. The *leftmost* well of both gels were loaded with 4 μL of a protein molecular weight standard (Precision Plus Protein™ Dual Xtra, Bio-Rad Laboratories). One well in Gel A was loaded with 500 fm of BSA standard; other three wells were loaded with 22 μg, 10 μg and 2 μg each of sheep serum protein sample. After the molecular weight standard, the other three wells in Gel B were loaded with 500 fm, 2500 fm and 250 fm each of BSA standard. *Arrows* show BSA standard

**Fig. 2:**
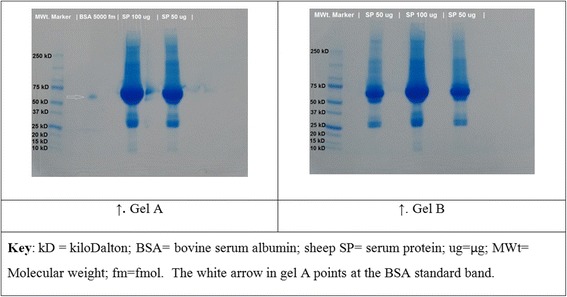
Coomassie-stained 1D SDS-PAGE gels used in the second in-gel digestion. Fractions of acetone precipitated serum protein samples from a healthy sheep were used. One well in Gel A was loaded with BSA standard (*arrow*), and two other wells were loaded with 100 μg and 50 μg of protein each. Three wells in Gel B were loaded with 50 μg, 100 μg and 50 μg each of protein

**Fig. 3:**
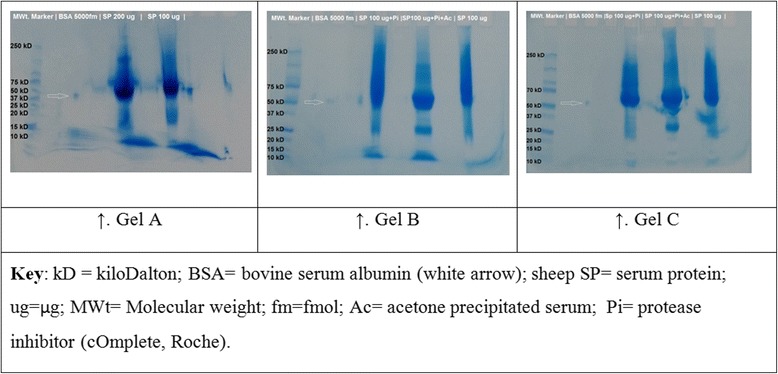
Coomassie-stained 1D SDS-PAGE gels used for the third in-gel digestion. Fractions of pooled serum protein samples from six healthy sheep were used. In Gel A, one well was loaded with 200 μg of crude serum protein and the other well was loaded with 100 μg. Three 100 μg of serum protein for Gel B and C were loaded identically and treated as follows: one well was loaded with crude serum with a protease inhibitor (SP 100 μg + Pi), the second well had acetone precipitated serum and a protease inhibitor (SP 100 μg + Pi + Ac); the third well was loaded with crude serum only (SP 100 μg)

The results of the first, second and third in-gel digestions are presented in Figs. 1, 2 and 3, respectively. The details of the individual gels are provided in the figure captions. Except for Gel B of in Fig. 1, the protein sample lanes of all the other gels were subjected to in-gel digestion followed by nanoLC-nanoESI-MS/MS to identify proteins. The protein ID results of the first, second and third in-gel and in-solution digestions are summarised in Table 1. The detailed results are presented in the accompanying spreadsheet Microsoft® Excel™ file [see Additional file 2]. Protein IDs were obtained using ProteinPilot™ [55] to search a UniProtKB composite database of *Ovis aries, Bos taurus and Capra hircus* with a results quality of FDR ≤1%; ≥ 2 peptides for a protein to be considered confidently identified as the highest scoring member of the protein group. The Pro Group™ Algorithm in ProteinPilot™ assigned one protein the best confidence possible (unused score) among protein isoforms, which enabled protein subset differentiation, as well the suppression of false positives for protein-grouping analysis [55]. The results were therefore based on protein group identifications presented as protein identifications (IDs).

**Table 1:** The number of proteins identified by ProteinPilot™ Software from in-gel and in-solution digestion of healthy sheep serum samples by searching a composite *Ovis aries, Bos taurus and Capra hircus* protein sequence database

Experiment →	First digestion		Second digestion		Third digestion	
Digestion type	In-gel	In-sol	In-gel	In-sol	In-gel	In-sol
Serum protein source	Ac	Ac	Ac	Crude	Ac + Crude	Crude
Total quantity of protein analysed	34 μg	10 μg	350 μg	20 μg	900 μg	100 μg
Number of protein IDs	120	25	241	100	182	32

Key: *In-sol* In-solution; *Ac* Acetone precipitated; *IDs* Identifications

In the present set of experiments, proteins were identified by using peptide signatures to search custom-built protein sequence databases. Protein ID confidence was determined by the number of proteins that were assuredly accepted as correct, having been identified as described elsewhere [56, 57]. Overall, a total of 267 confident and unique protein groups were identified using ProteinPilot™ by searching a composite *Ovis aries, Bos taurus and Capra hircus* UniProtKB database after combining all the three in-gel digestion workflows (first, second and third in-gel digestions) from a total quantity of 1,284 μg of serum protein obtained from six healthy sheep. The UniProtKB entries for the identified proteins are presented in Additional file 2.

### In-solution digestion

A composite ProteinPilot™ search of all the three in-solution digestion workflow samples comprising of 130 μg of serum protein yielded a total of 102 protein IDs. The UniProtKB entries for these proteins are presented in Additional file 2.

**Fig. 4:**
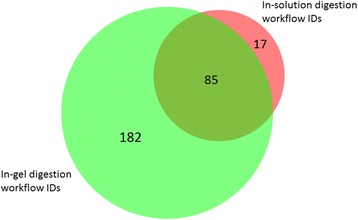
Comparison of lists of protein identifications (IDs) derived from in-solution versus in-gel digestion using BioVenn Software [51]. Proteins were identified by searching a composite database of *Bos taurus, ovis aries and Capra hircus* using ProteinPilot™ Software. Only 17 protein IDs were exclusive to in-solution workflow compared to 182 protein IDs exclusive to in-gel workflow

A comparison between the protein identification list derived from combined first, second and third in-gel digestion (in-gel digestion workflow) and that of combined first, second and third in-solution digestion (in-solution digestion workflow) in BioVenn Software [51] is presented in Fig. 4. The UniProtKB entries of the 17 proteins that were exclusive to the in-solution digestion workflow (i.e. proteins were not detected by in-gel workflow) are A0A0F6QNP7, W5PSQ7, W5QH45, W5NQW9, G5E604, W5PZF0, W5NWX6, Q1KZF3, W5PJZ2, W5QDP8, W5PDR7, W5PN97, W5PXI6, F1N3Q7, C6ZP49, G3N346 and Q3SYR8.

A combined ProteinPilot™ search of the pilot data from one sheep and the additional data from five sheep for both in-gel and in-solution digestion workflows using a composite *Ovis aries, Bos taurus and Capra hircus* protein sequence database yielded an overall outcome of 274 protein IDs. The details of these protein IDs and their peptide sequences are presented in table format in Additional file 2. Based on comparison with previous studies and protein database resources [10, 14, 15, 24, 25, 58–67], a table was drawn from the preceding references listing the details of 67 known, 207 novel and 83 disease-associated serum proteins identified is presented as Additional file 3. The known proteins are those that have been cited in the literature and also have a confirmed status in UniProtKB. Novel proteins constitute those that previously appeared as predicted and proteins that had hitherto been inferred by homology. Disease-associated proteins refer to proteins that are expressed or alter during pathology in sheep and other species.

### Combined protein identifications from 1D SDS-PAGE and in-solution digestion of serum using ProteinPilot™ and Mascot database search engines and PeptideShaker search

Protein yields of a composite search of all the sample data from the three workflows (first, second and third in-gel and in-solution digestion) using a sheep-only UniProtKB database optimised for PeptideShaker Software were as follows: ProteinPilot™: 245 IDs and Mascot: 379 IDs. A secondary analysis using PeptideShaker of this same entire dataset yielded 133 IDs (1% FDR and ≥ 2 unique validated peptides). The details of these protein IDs are provided in Additional file 4. Again, based on comparison with previous studies and protein database resources [10, 14, 15, 24, 25, 58–67], and using the 379 Mascot protein IDs, a table was drawn from the preceding references to list the details of 77 known, 302 novel and 83 disease-associated serum proteins identified using this sheep only database in Additional file 5.

The 379 protein IDs from Mascot search were used as a benchmark for further downstream analysis. Every sheep protein ID made in Mascot was mapped to a distinct gene. Of all the 379 protein IDs made by searching the sheep-only UniProtKB database, only 74 proteins had been annotated based on sequence similarity to other species, whilst 305 proteins were uncharacterised. Of the 74 annotated proteins, only annexin A2 (P14639), serum albumin (P12303), transthyretin (B3SV56), nuclear receptor subfamily 1 group D member 1 (A2SW69) and insulin-like growth factor-binding protein 2a (Q29400) had been reviewed and therefore included in the Swiss-Prot subset of UniProtKB. The unreviewed, but named proteins included apolipoprotein E, fibulin-1, angiotensinogen, monocyte differentiation antigen CD14, plasminogen, pentaxin (pentraxin), alpha-1-antitrypsin transcript variant 1, histone H2B, alpha-1-acid glycoprotein, amine oxidase, beta-A globin chain, thyroxine-binding globulin, alpha-2-HS-glycoprotein, C-X-C motif chemokine, histone H3, coagulation factor IX, histone H4, factor H, prothrombin, clusterin, L-lactate dehydrogenase, cGMP-dependent protein kinase, antithrombin-III, gelsolin isoform b, ceruloplasmin, VH region chain, conglutinin 1, DNA polymerase, proteasome subunit alpha type, tubulin beta chain, proteasome subunit alpha type, proteasome subunit alpha type, fibrinogen alpha chain, aspartate aminotransferase, phosphodiesterase, chitinase-3-like protein 1, superoxide dismutase [Cu-Zn], uricase, glyceraldehyde-3-phosphate dehydrogenase, carbonic anhydrase 2, adiponectin, olfactory receptor, histone H2A, alpha-mannosidase, centromere protein C, importin subunit alpha, 14-3-3 protein sigma, AP complex subunit beta, carboxypeptidase, oxysterol-binding protein, growth hormone receptor variant H, condensin complex subunit 2, large tumour suppressor-like 1 protein,protein-tyrosine-phosphatase, peptidyl-prolyl cis-trans isomerase (PPIase), proteasome subunit alpha type, dipeptidase, proteasome subunit beta type, tubulin alpha chain, proteasome subunit alpha type, fructose-1,6-bisphosphatase 1, polypeptide N-acetylgalactosaminyltransferase, arginase, adenylyl cyclase-associated protein, protein-serine/threonine kinase, transaldolase, MHC class II antigen, glutathione peroxidase and corneodesmosome protein.

### **Gene ontology (GO)** – **term analysis of proteins identified in serum of healthy sheep**

The 379 proteins identified by a composite Mascot search of the first, second and third in-gel and in-solution digestion of serum proteins from healthy sheep were subjected to gene ontology (GO) analysis using Protein ANalysis THrough Evolutionary Relationships (PANTHER) classification tool [68]. In the PANTHER tool, the gene entries were analysed by aligning them to *Bos taurus* as the closest organism analogous to sheep because *Ovis aries* entries were not available. The PANTHER analysis resulted into 349 bovine aligned gene entries listed in Additional file 6.

**Fig. 5:**
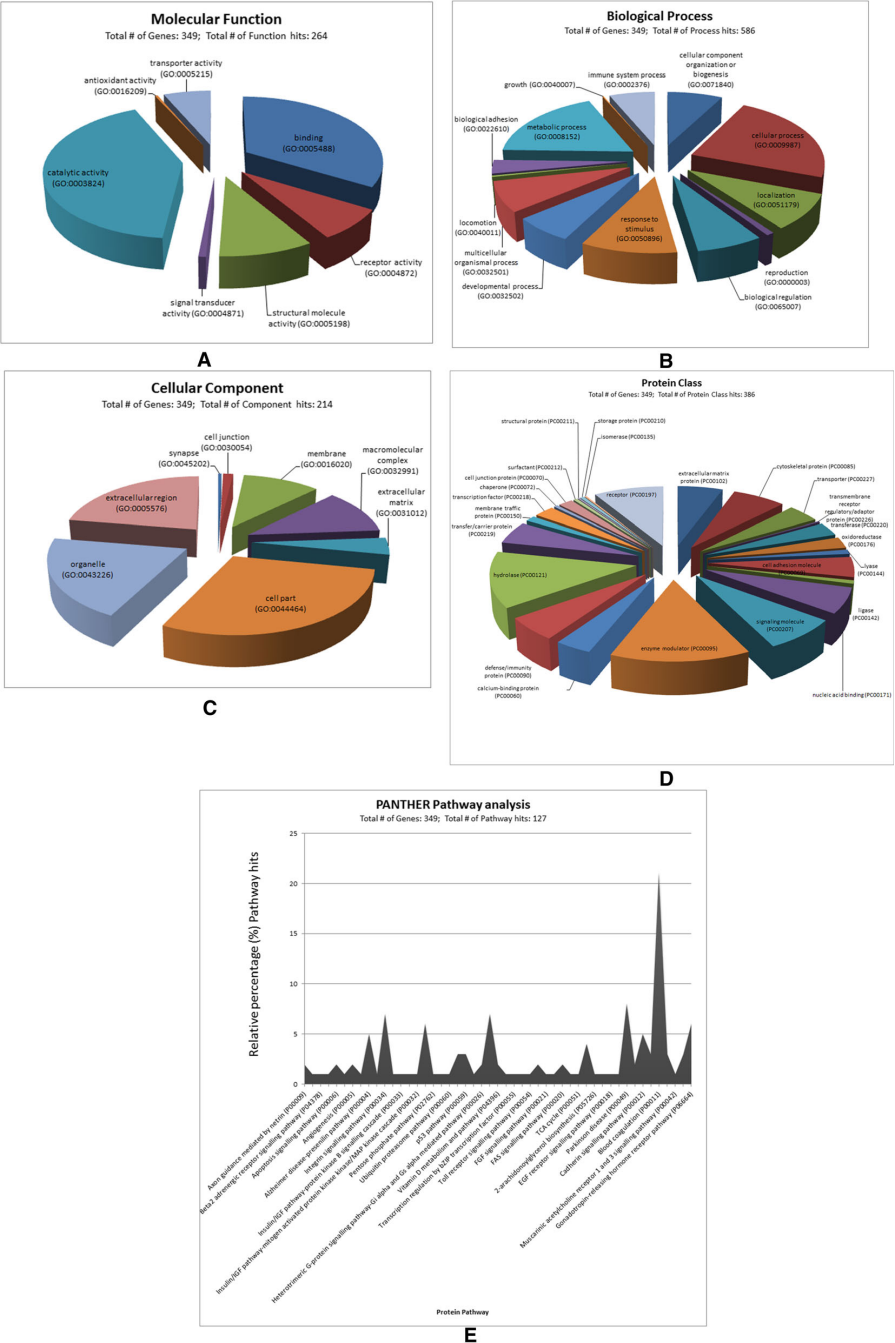
Gene Ontology (GO) terms and pathway analysis of sheep proteins identified by Mascot database search engine. Protein data were derived from combined in-gel and in-solution workflows of serum protein samples from healthy sheep aligned to *Bos taurus* gene entries. Up to 349 genes were resolved from a total number of 379 sheep protein identifications (IDs) by the Protein Analysis THrough Evolutionary Relationships (PANTHER) classification tool [68]. The GO domain of molecular function had 264 function hits (**a**); biological process had 586 process hits (**b**); cellular component had 214 component hits (**c**); protein class had 386 class hits (**d**). There were 127 protein pathway hits with 49 prominent pathways, but only 25 are displayed (**e**). The other 24 protein pathway names not displayed with their codes and percentage contribution in parenthesises were: Axon guidance mediated by Slit/Robo (P00008)(0.80%), Beta1 adrenergic receptor signalling pathway (P04377)(0.80%), JAK/STAT signalling pathway (P00038) (0.80%), Ionotropic glutamate receptor pathway (P00037)(0.80%), Alzheimer disease-amyloid secretase pathway (P00003)(0.80%), Phenylethylamine degradation (P02766)(0.80%), Phenylalanine biosynthesis (P02765)(0.80%), Inflammation mediated by chemokine and cytokine signalling pathway (P00031)(4.70%), Asparagine and aspartate biosynthesis (P02730)(0.80%), Huntington disease (P00029)(2.40%), Heterotrimeric G-protein signalling pathway-Gq alpha and Go alpha mediated pathway (P00027)(0.80%), Wnt signalling pathway (P00057)(5.50%), Glycolysis (P00024)(0.80%), General transcription regulation (P00023)(0.80%), T cell activation (P00053)(1.60%), TGF-beta signalling pathway (P00052)(0.80%), Tyrosine biosynthesis (P02784) (0.80%), Plasminogen activating cascade (P00050) (3.10%), Endothelin signalling pathway (P00019)(0.80%), DNA replication (P00017)(0.80%), Cytoskeletal regulation by Rho GTPase (P00016)(1.60%), Nicotinic acetylcholine receptor signalling pathway (P00044)(2.40%), B cell activation (P00010)(2.40%) and CCKR signalling map (P06959)(2.40%)

The results of GO-term analysis of molecular function, biological process, cellular component, protein class and pathway analysis of the detected proteins are provided in Fig. 5. Looking at the molecular function domain of the proteins alone based on the GO term results (Fig. 5a), catalytic activity was dominant of the 264 function hits. From the protein IDs that had names, at least 27 of them were specifically classified as enzymes from protein database searches. It is evident from these results that there is a hierarchy in the biological processes of the 586 process hits (Fig. 5b). The cellular component GO domain (Fig. 5c) for serum from healthy sheep had 214 hits in total. The protein class GO domain (Fig. 5d) had 386 class hits, with enzyme modulation topping the list. Among the 49 prominent protein pathways that were displayed in PANTHER from the analysed genes, 14 were represented by over 3.0% contribution to the revealed pathway pool (Fig. 5e).

## Discussion

This study reports the development of a proteomics baseline profile of healthy sheep serum by analysing peptides derived from in-solution digestion and 1D SDS-PAGE using nanoLC-nanoESI-MS/MS. The major outcome was that 379 proteins were identified, compared for example to 42 proteins from serum of sheep with mild respiratory disease during peripartum period [10] and a single protein (serum amyloid A) in sheep with scrapie [15]. Both of these cited earlier sheep studies used two dimensional (2-DE) surface enhanced laser desorption/ionisation time of flight mass spectrometry (SELDI-TOF MS) and LC-MS/MS. In species other than sheep, 490 proteins were identified in human sera using multidimensional separation coupled with MS [2], while 340 low molecular weight proteins were identified in human sera using SELDI-TOF MS analysis and LC-MS/MS [69]. There is also a report that assessed three different lots of foetal bovine serum by NanoLC-MS/MS analysis in which 79, 90, and 91 proteins were identified [70]. The preceding study recognised that there is variability in the protein content of different lots of foetal bovine serum – a commonly used growth medium for cell cultures, which affects the consistency of cell growth. The lot with a higher number of protein IDs was associated with higher cell growth rate [70]. Identification of these proteins is important clinically to determining health or altered physiology, such as stress [10].

The use of 1D SDS-PAGE in this study facilitated serum protein samples to be fractionated to reduce protein complexity prior to nanoLC-nanoESI-MS/MS analysis [71]. The first in-gel digestion experiment enabled the determination of the quantity of protein from samples and the amount of the BSA standard that needed to be loaded onto the gel to ensure that protein bands were visible and clearly defined (Fig. 1). Loading a larger quantity of protein onto the gel was necessary to discover as many proteins as possible using DDA [72]. However, the 2 μg lane yielded 41 protein IDs in the first in-gel digestion (Fig. 1), while the 10 μg-lane yielded 20 protein IDs and the 22 μg lane yielded 121 protein IDs. The 10 μg lane was analysed initially and the 2 μg and 22 μg lanes were analysed 6 weeks later once the extractions had been optimised and the instrument tuned.

The second in-gel digestion (Fig. 2) increased the protein coverage by loading more protein into the gel wells using a fraction of the acetone precipitated serum sample used in the 1^st^ in-gel digestion. The 100 μg (2 replicates) and 50 μg (3 replicates) protein loads in the 2^nd^ in-gel digestion workflow yielded comparable numbers of protein IDs for each of the loaded quantity of protein. This suggests that reproducibility of the amount of protein loaded into the gel lanes had been achieved [71]. The second in-gel digestion was an improvement of the 1st in-gel digestion by having replicate and having increased quantities of loaded protein per lane, using the same serum sample of 1st in-gel digestion from Sheep ID 473.

The 1D SDS-PAGE preparation of one gel in the third in-gel digestion had a number of visual artefacts (Fig. 3). The distortion in the 10-15kD region of Gel A could have been attributed to a defect in the gel possibly due to inconsistency in gel polymerisation creating artefact bands [35], overloading and/or the presence of a pocket between the gel and the cassette housing that allowed the protein samples to leak out the gel [73]. This could have also contributed to the low number of protein yields made from this gel (200 μg: 40 protein IDs; 100 μg: 38 protein IDs), compared to the 100 μg × 2 lanes in Gels B and C that yielded 114 protein IDs [see Additional file 2]. A couple of variables were also introduced in this experiment, in addition to the quantity of proteins loaded on to the gel wells as planned. The analysis of fractionated crude serum that had a protease inhibitor (cOmplete, Roche) yielded a higher number of protein IDs (162 IDs), compared to the acetone precipitated sample that also had the protease inhibitor (143 IDs). This suggests that a considerable number of proteins were present in the acetone precipitation supernatant that was discarded. The discardment of the supernatant from acetone precipitation is a routine practice during generic or universal sample preparation for proteomic analysis [74].

As for the in-solution digestion workflow, the number of protein identifications from analysing 100 μg of crude serum protein was low when compared with 20 μg. The sample for the first in-solution digestion using 10 μg of acetone precipitated serum that was drawn from one healthy pilot sheep (Sheep ID 473) yielded only 25 protein IDs. This sample was prepared and analysed at the same time as the 10 μg sample of the first in-gel digestion discussed earlier. Protein detection was therefore likely to have been affected by unoptimised experimental processes at the time prior to running on the MS instrument. The second in-solution digestion using 20 μg of crude serum from the same sheep yielded 100 protein IDs. This result was considered substantial, as the number of protein IDs was comparable to those of other studies [10, 75–81]. Unexpectedly however, the third in-solution that utilised 100 μg of pooled crude serum from six sheep under the same experimental conditions yielded only 32 IDs. It is thought that this result was possibly due to the inhibition of trypsin by the presence of intravenous agents in the pooled sample from the anaesthetic cocktail used to anaesthetise the sheep, as this was not the case with the pilot sheep sample in which the sheep was not anaesthetised during sample collection.

BioVenn Software [51] was utilised for visualisation of the data presented in Fig. 4. This tool enabled the comparison of a protein identification list derived from in-gel digestion with that from in-solution digestion by displaying the data in an area-proportional Venn diagram. It showed protein IDs that were exclusive to in-solution and in-gel, and those common between the two digestions. The composite in-solution digestion workflow yielded 102 protein IDs. Of the 17 protein IDs that were exclusive to in-solution digestion workflow, five were mapped to the ox, two to the goat and the remaining 10 IDs were for sheep. Despite having known genes, the vast majority of the identified proteins were either uncharacterised or unreviewed in UniProtKB. Another interesting observation was that the combined list of 284 protein IDs from in-gel and in-solution digestion displayed in BioVenn Software was marginally higher than the 274 IDs from a composite ProteinPilot™ search of the same datasets. It is likely that the subsequent composite ProteinPilot™ search helped to further group proteins, thereby improving the confidence of protein IDs by minimising false protein identifications – a known challenge when searching a multi-species protein database to identify proteins.

A combined search of the first, second and third in-gel and in-solution digestion datasets using a sheep-only database yielded 245 protein IDs in ProteinPilot™ (*cf* 274 protein IDs using the composite database of the ox, goat and sheep) and Mascot search yielded 379 IDs. The PeptideShaker validation search yielded 133 protein IDs. The comparatively low number of protein IDs made by PeptideShaker is because the protein entries were identified using validated unique peptides – a feature that is not obvious in either ProteinPilot™ or Mascot, whose protein ID entries were only based on at least two high-scoring peptides per protein, on the assumption that the peptides were unique to the protein.

The results from Mascot search were embraced and utilised for further analysis because this software platform is widely used by the proteomics community and it is considered the industry standard, as it implements a vast array of applications necessary for protein identification [82]. As of September, 2016, the 379 protein IDs complete with UniProtKB accessions was probably the highest number of sheep serum proteins to date using nanoLC-nanoESI-MS/MS. Of these protein IDs, only 74 were named in UniProtKB, whilst the vast majority (305) were yet to be characterised. This study can therefore be considered the first to provide a comprehensive MS/MS protein sequence data of serum proteins of normal sheep and by contributing to the efforts of annotating genes and charactering sheep proteins. Despite most of the proteins not being characterised in UniProtKB, their mapping to known genes and the available mass spectrometry-derived peptide sequence data alongside verification on more than one software platforms, constitute strong supportive evidence that the identified proteins do exist. The downside of the Mascot search is that it does not provide a user-friendly protein sequence output that can be readily tabulated as in the case of ProteinPilot™ IDs. For this reason, only protein names and UniProtKB entries were utilised mostly for the purposes the present study.

Regarding GO-term analysis, the significance of many of the enzymes that dominated catalytic activity in the molecular function domain (Fig. 5 a), remains to be documented in sheep, but the functions of some are known. For example, adenylyl cyclase-associated protein regulates cofilin function, actin cytoskeleton and cell adhesion [83]. Alpha-mannosidase participates in glycoprotein synthesis and endoplasmic reticulum quality control [84]. It has been reported to be downregulated in locoweed *(Oxytropis sericea)* in sheep [85, 86], for example. The functions of other identified enzymes that were drawn from [24, 87–116] are provided in Additional file 7.

Serum samples of healthy adult female Merino sheep were utilised for this study. It is quite possible that a relatively low representation of the growth process domain in the biological process GO-term was because serum samples were derived from adult sheep. Also, the cellular component fractions could possibly vary depending on the physiological status of the sheep – which remains yet to be determined and documented. It can be argued that hormonal changes and the influence of age contribute to observations of serum proteome profiles and this should be accounted for. For instance, studies in sheep have shown that there is a diurnal variation metabolic and stress-responsive hormones [117].

In the present study, there were mechanisms in place to mitigate the effects of stress on the laboratory sheep. The sheep were reared together and acclimatised to their housing and handling by people as a standard management practice prior to blood sampling [33, 118]. Also, there was no variation in calorie intake because feed was supplemented as required [33, 118] in order to mitigate the well-established phenomenon of seasonal weight loss – a well-established major nutritional stress factor in sheep [119]. During agistment, there were wethers that belonged to other experiments of the research group, but there were no entire males to cause ‘ram effect’ that could have caused surges in reproductive hormones [120], for example. Nevertheless, gonadotropic activity would have occurred naturally in the ewes to cause hormonal changes [121], perhaps even with a synchronised hypothalamic-pituitary-ovarian axis in all the ewes, as this phenomenon is known to occur naturally [122]. All the sheep were approximately 2 years old and were therefore, practically in the same metabolic and physiological state during blood sampling. Also, the sheep belonged to an ovine model of blood transfusion [123], so most preventable adverse attributes had been catered for.

The fundamental ‘method’ for pulling proteins from the liquid fraction of blood using the explored approach is already well-developed in itself, but this study went beyond this to develop a tailored platform, comprising a series of refined methods, to give this practical application. The knowledge from this prototype study has illuminated a considerable number of bovine-aligned gene entries associated with protein pathways that can be valuably exploited by animal model studies using sheep serum as their analyte. A downside of the present study is that no males were represented in the dataset. Future studies should take into account hormonal changes, be gender and age inclusive in order to capture broad aspects of the proteome that could have been missed in this report.

## Conclusion

This study has demonstrated for the first time that it is feasible to identify several hundred sheep serum proteins using nanoLC-nanoESI-MS/MS. By utilising the PANTHER tool, this serum-derived prototype of the ovine circulating acellular proteome revealed association of 349 genes with 127 protein pathway hits. When used with protein quantitative data, these findings have the potential to be applied as the foundation for establishing the baseline normal ovine serum proteome that could be used in comparison with samples from sick sheep. The peptide spectral data here also are a contribution towards a library that can be applied for targeted proteomics approaches, such as sequential acquisition of all theoretical fragrant mass spectra (SWATH)-MS to fulfil proteogenomics study efforts on sheep in future.

## Additional files

Additional file 1: One-dimensional sodium dodecyl sulfate polyacrylamide gel electrophoresis (1D SDS-PAGE). In-gel fractionation (1D SDS-PAGE) of sheep serum protein samples. (DOCX 24 kb)

Additional file 2: Protein identification results from using ProteinPilot™ to search a composite *(Bos taurus, Ovis aries and Carpra hircus)* UniProtKB protein sequence database of serum samples derived from the first, second and third in-gel and in-solution digestion with a results quality of FDR ≤1%; ≥ 2 peptides for the highest scoring member of the protein group to be considered confidently identified. Each tab contains a list of protein IDs based on the quantity of protein loaded (μg), digestion workflow or sample conditions as follows: 1st_In-gel_digestion_2 μg = first in-gel (2 μg); 1st_In-gel_digestion_10 μg = first in-gel (10 μg); 1st_In-gel_digestion_22 μg = first in-gel (22 μg); All_1st_In-gel_digestion_IDs = all first in-gel samples; 2nd_In-gel_digestion_100 μgGelA = second in-gel digestion of Gel A (100 μg); 2nd_In-gel_digestion_50 μgGelA = second in-gel digestion of Gel A (50 μg); 2nd_In-gel_digestion_50 μgGelB = second in-gel digestion of Gel B (50 μg); 2nd_In-gel_digestion_100μgGelB = second in-gel digestion of Gel B (100 μg); 2nd_In-gel_digestion_50μgGelB = second in-gel digestion of Gel B (50 μg); All_2nd_In-gel_digestion_IDs = composite of all second in-gel digestion samples; 3rd_In-gel_digestion_200μg = crude serum protein of the third in-gel digestion (200 μg); 3rd_In-gel_digestion_100μg = crude serum protein of the third in-gel digestion (100 μg); 3rd_In-gel_digest_100μgx2CrudeI = crude serum protein with a protease inhibitor (Roche) of the third in-gel digestion (100 μg × 2); 3rd_In-gel_digest_100μgx2AcePPT = acetone precipitated serum protein without a protease inhibitor of the third in-gel digestion (100 μg × 2); 3rd_In-gel_digest_100μgx2No_I = crude serum protein without a protease inhibitor of the third in-gel digestion (100 μg × 2); All_3rd_In-gel_Digestion_IDs = composite of all third in-gel digestion samples; All_In-gel_digestion_IDs = all in-gel digestion workflow; 1st_In-solution_digestion_10 μg = acetone precipitated serum protein from the first in-solution digestion (10 μg); 2nd_In-solution_digestion_20 μg = crude serum protein from the second in-solution digestion (20 μg); 3rd_In-solution_digestion_100μg = crude serum protein from the third in-solution digestion (100 μg); All_In-solution_digestion_IDs = all in-solution workflow samples; All_Proteins + Peptide_Sequences = 274 protein IDs and peptide sequences of the entire in-gel and in-solution digestion experiments. (XLSX 423 kb)

Additional file 3: Details of known, novel and disease-associated sheep serum proteins identified by ProteinPilot™ by a searching a composite UniProtKB protein sequence database *of Bos taurus, Ovis aries and Capra hircus.* This 3-sheet Microsoft Excel file contains the details of 67 known (Known_Proteins_in_Literature), 207 novel (Novel_Proteins) and 83 disease-associated (Disease-Associated_Proteins) serum proteins identified using this composite database. The known proteins are those that have been cited in the literature and also have a confirmed status in UniProtKB. Novel proteins constitute those that previously appeared as predicted and proteins that had hitherto been inferred by homology. Disease-associated proteins refer to proteins that are expressed or alter during pathology in sheep and other species. (XLSX 161 kb)

Additional file 4: Protein identifications by ProteinPilot™, Mascot and PeptideShaker search engines from searching a composite of the first, second and third digestions sheep serum protein data using an *Ovis aries* only UniProtKB protein sequence database. This 3-sheet Microsoft® Excel™ file contains the details of protein identifications of a composite search of all the sample data from the three workflows (first, second and third in-gel and in-solution digestion) using an *Ovis aries* only UniProtKB database optimised for PeptideShaker Software. The tab details are as follows: ProteinPilot_IDs_ + _Sequences = 245 ProteinPilot™ protein IDs complete with peptide sequences; Mascot_IDs = 379 Mascot protein IDs and PeptideShaker_IDs = 133 PeptideShaker protein IDs. (XLSX 170 kb)

Additional file 5: Details of known, novel and disease-associated sheep serum proteins identified by Mascot from searching a composite of the first, second and third digestions sheep serum protein data using an *Ovis aries* UniProtKB protein sequence database. This 3-sheet Microsoft Excel file contains the details of 77 known (Known_Proteins_in_Literature), 301 novel (Novel_Proteins) and 83 disease-associated (Disease-Associated_Proteins) serum proteins identified using an *Ovis aries* UniProtKB database. (XLSX 14 kb)

Additional file 6: List of 349 bovine aligned gene entries derived from inputting 379 *Ovis aries* protein data in the PANTHER classification tool. This 2-sheet Microsoft Excel file contains the list of 349 bovine aligned gene entries (349_Bovine_aligned_gene_entries) derived from analysing gene information of 379 serum proteins identified by Mascot (Input_of_379_Ovine_Mascot_IDs) using an *Ovis aries* UniProtKB database. (XLSX 49 kb)

Additional file 7: Functions of dominant enzymes identified in healthy sheep serum. The functions of enzymes identified in healthy sheep serum that dominated catalytic activity in the molecular function domain after gene ontology analysis. (DOCX 67 kb)

## Abbreviations

1D SDS-PAGE: One-dimensional sodium dodecyl sulfate polyacrylamide gel electrophoresis
ACN: Acetonitrile
ARCBS: Australian Red Cross Blood Service
BCA: Bicinchoninic acid assay
BSA: Bovine serum albumin
CARF: Central Analytical Facility
DDA: Data dependent acquisition
DNA: Deoxyribonucleic acid
DTT: Dithiothreitol
EDTA: Ethylenediaminetetraacetic acid
ESI-QUAD-TOF: Electrospray ionisation quadrupole time-of-flight
FA: Formic acid
FDR: False discovery rate
GO: Gene ontology
IAM: Iodoacetamide
ID: Identification
LC: Liquid chromatography
MGRF: Molecular Genetics Research Facility
MHC: Major histocompatibility complex
MS: Mass spectrometry
MS/MS: tandem mass spectrometry
nanoLC-nanoESI-MS/MS: nano liquid chromatography nano electrospray ionisation tandem mass spectrometry
NCBI: National Center for Biotechnology Information
PANTHER: Protein analysis through evolutionary relationships
PRIDE: Proteomics identifications
QUT: Queensland University of Technology
RT: Room temperature
SP: Serum protein
SWATH: Sequential acquisition of all theoretical fragrant mass spectra
TEMED: Tetramethylethylenediamine
TFA: Trifluoroacetic acid
TOF: Time-of-flight
UQ: The University of Queensland
