# Supplementary material for: Characterisation of the circulating acellular proteome of healthy sheep using LC-MS/MS-based proteomics analysis of serum
Source: Proteome Sci. 2017 Jun 10;15:11. doi: 10.1186/s12953-017-0119-z (PMC5466729; doi:10.1186/s12953-017-0119-z)
Supplement: Supplementary file 1 — One-dimensional sodium dodecyl sulfate polyacrylamide gel electrophoresis (1D SDS-PAGE). In-gel fractionation (1D SDS-PAGE) of sheep serum protein samples. (DOCX 24 kb) [file 12953_2017_119_MOESM1_ESM.docx]

**One-dimensional sodium dodecyl sulfate polyacrylamide gel electrophoresis (1D SDS-PAGE) of sheep serum protein samples.**

The universal 1D SDS-PAGE procedure adapted for protein separation (also referred to as in-gel separation of proteins) was based on its established description [[29](#_ENREF_29)] and subsequent refinements [[30-33](#_ENREF_30)]. Briefly, the resolving self-cast 10% SDS-PAGE gels were prepared by mixing 3.85 mL of deionised water (Millipore) with 2 mL of 40% acrylamide/ 2% bisacrylamide mix, 2 mL of 1.5M tris (pH 8.8), 80 µL of 10% SDS, 80 µL of 10% ammonium persulfate and 8 µL of Tetramethylethylenediamine (TEMED, Bio-Rad Laboratories), which was then poured into a casting chamber (Mini-PROTEAN, Bio-Rad Laboratories) and left to set after overlaying the gel with isopropanol. The isopropanol was then removed and the stacking 4% SDS-PAGE gels were prepared by mixing 3.15 mL of deionised water (Millipore) with 0.5 mL of 40% acrylamide/bisacrylamide, 2 mL of 0.5 M tris (pH 6.8), 50 µL of 10% SDS, 50 µL of 10% ammonium persulfate and 5 µL of TEMED, and then poured on top of the resolving gel followed by insertion of plastic comb to mould the loading wells. The gels were left to polymerise for 12 h at room temperature (RT) before loading the wells with known quantities of protein samples. A stock solution of 10× running buffer was prepared by mixing 30 g of Tris, 144 g of glycine, 10 g of SDS and made up to 1L solution using de-ionised water (Millipore) before storage at RT. A 5× sample buffer stock was prepared by mixing 1.75 mL of 0.5M Tris (pH 6.8), 4.5 mL of glycerol, 0.5 g of SDS, 1 drop of 2.5mg/mL solution of bromophenol blue. The solution was brought up to 10 mL, then aliquoted into 500 uL and stored at -20^o^C until use.

To load the gel, a desirable quantity of protein in the sample was aimed at approximately 0.1 µg/µL for each stained protein band [[31](#_ENREF_31)] in either self-cast or precast polyacrylamide gel (Mini-PROTEAN® TGX™ Precast Gels, Bio-Rad Laboratories). A protein standard (Precision Plus Protein™ Dual Xtra, Bio-Rad Laboratories) was loaded into to the first well of every gel for the estimation of the protein molecular weight. Protein concentrations of bovine serum albumin (BSA) standard (Pierce) and the samples were adjusted so that suitable amounts of protein could be loaded onto the gel with freshly prepared 20mM solution of DTT in LC/MS-grade water with 1× SDS sample buffer. Gel loading preparations were made in 1,500 µL (or 500 µL, where appropriate) Eppendorf tubes and then vortexed briefly before being centrifuged at 4,000 *g* for 6 s. The tubes containing the sample buffer and sample were heated at 56 ^o^C for 3 min to unfold proteins prior to loading onto the gel and electrophoretic separation of proteins at a fixed voltage (150-180V) for approximately 45 min in a vertical electrophoresis chamber (Mini-PROTEAN Electrophoresis Cell, Bio-Rad Laboratories) powered by PowerPac™ Universal power supply from Bio-Rad Laboratories.

The gels were stained with Coomassie brilliant blue (EZ-Run™, Protein Gel Staining Solution, Fisher Scientific) according to the manufacturer’s instructions and then photographed using a handheld camera (5.7-inch Quad HD Super AMOLED^®^, Samsung; or New 8-megapixel iSight camera with 1.5µ pixels with Optical image stabilisation, iPhone 6, Apple Inc.).
