# Supplementary material for: Characterisation of the circulating acellular proteome of healthy sheep using LC-MS/MS-based proteomics analysis of serum
Source: Proteome Sci. 2017 Jun 10;15:11. doi: 10.1186/s12953-017-0119-z (PMC5466729; doi:10.1186/s12953-017-0119-z)
Supplement: Supplementary file 7 — Functions of dominant enzymes identified in healthy sheep serum. The functions of enzymes identified in healthy sheep serum that dominated catalytic activity in the molecular function domain after gene ontology analysis. (DOCX 67 kb) [file 12953_2017_119_MOESM7_ESM.docx]

**Additional file 7. Functions of dominant enzymes identified in healthy sheep serum**

| **Enzyme** | **Function** | **Reference/s** |
| --- | --- | --- |
| Amine oxidase | Deamination of aliphatic amines. | [[86](file:///C:\Users\Jude\AppData\Local\Temp\PSCI-D-16-00055R1%20v6.docx#_ENREF_86)] |
| Arginase | Associated with collagen deposition during acute lung injury and *Pseudomonas aeruginosa* infection. | [[87](file:///C:\Users\Jude\AppData\Local\Temp\PSCI-D-16-00055R1%20v6.docx#_ENREF_87)] [[88](file:///C:\Users\Jude\AppData\Local\Temp\PSCI-D-16-00055R1%20v6.docx#_ENREF_88)] |
| Aspartate aminotransferase | Well-established in veterinary medicine as part of liver function test profiling and other pathology such as mastitis in sheep | [[89](file:///C:\Users\Jude\AppData\Local\Temp\PSCI-D-16-00055R1%20v6.docx#_ENREF_89)] [[90](file:///C:\Users\Jude\AppData\Local\Temp\PSCI-D-16-00055R1%20v6.docx#_ENREF_90)] |
| Carbonic anhydrase 2 | Regulates ammonia, CO_2_ and magnesium in the rumen. | [[91](file:///C:\Users\Jude\AppData\Local\Temp\PSCI-D-16-00055R1%20v6.docx#_ENREF_91)], [[92](file:///C:\Users\Jude\AppData\Local\Temp\PSCI-D-16-00055R1%20v6.docx#_ENREF_92)] |
| Carboxypeptidase | Oxidative stress response and galactosialidosis. | [[93](file:///C:\Users\Jude\AppData\Local\Temp\PSCI-D-16-00055R1%20v6.docx#_ENREF_93)] [[94](file:///C:\Users\Jude\AppData\Local\Temp\PSCI-D-16-00055R1%20v6.docx#_ENREF_94)] |
| cGMP-dependent protein kinase | Multipurpose signalling enzyme. | [[95](file:///C:\Users\Jude\AppData\Local\Temp\PSCI-D-16-00055R1%20v6.docx#_ENREF_95)] |
| Chitinase-3-like protein 1 | Mediates the translocation of bacteria across epithelial membranes in mice with compromised epithelium. | [[96](file:///C:\Users\Jude\AppData\Local\Temp\PSCI-D-16-00055R1%20v6.docx#_ENREF_96)] |
| Dipeptidase | Cleaves leukotriene D4 and cystinyl-bis-glycine preventing bronchoconstriction. | [[97](file:///C:\Users\Jude\AppData\Local\Temp\PSCI-D-16-00055R1%20v6.docx#_ENREF_97)] |
| deoxyribonucleic acid polymerase | catalyses the formation of DNA from nucleotides | [[98](file:///C:\Users\Jude\AppData\Local\Temp\PSCI-D-16-00055R1%20v6.docx#_ENREF_98)] |
| Fructose-1,6-bisphosphatase 1 | Well-recognised role in carbohydrate metabolism and regulation of appetite | [[99](file:///C:\Users\Jude\AppData\Local\Temp\PSCI-D-16-00055R1%20v6.docx#_ENREF_99)] [[100](file:///C:\Users\Jude\AppData\Local\Temp\PSCI-D-16-00055R1%20v6.docx#_ENREF_100)] |
| Glutathione peroxidase | Harvests reactive oxygen species thereby preventing oxidative damage | [[101](file:///C:\Users\Jude\AppData\Local\Temp\PSCI-D-16-00055R1%20v6.docx#_ENREF_101)] |
| Glyceraldehyde-3-phosphate dehydrogenase | Involved in glycolysis | [[102](file:///C:\Users\Jude\AppData\Local\Temp\PSCI-D-16-00055R1%20v6.docx#_ENREF_102), [103](file:///C:\Users\Jude\AppData\Local\Temp\PSCI-D-16-00055R1%20v6.docx#_ENREF_103)] |
| L-lactate dehydrogenase | Oxidoreductase activity and a marker for subclinical mastitis in sheep | [[90](file:///C:\Users\Jude\AppData\Local\Temp\PSCI-D-16-00055R1%20v6.docx#_ENREF_90)] |
| Peptidyl-prolyl cis-trans isomerase | Accelerates the folding of proteins | [[23](file:///C:\Users\Jude\AppData\Local\Temp\PSCI-D-16-00055R1%20v6.docx#_ENREF_23)] |
| Phosphodiesterase | Splicing of nucleotides | [[104](file:///C:\Users\Jude\AppData\Local\Temp\PSCI-D-16-00055R1%20v6.docx#_ENREF_104)] |
| Plasminogen | Fibrinolysis and inflammation | [[23](file:///C:\Users\Jude\AppData\Local\Temp\PSCI-D-16-00055R1%20v6.docx#_ENREF_23)] |
| Polypeptide N-acetylgalactosaminyltransferase | Initiates the transformation of glycans into antigens | [[105](file:///C:\Users\Jude\AppData\Local\Temp\PSCI-D-16-00055R1%20v6.docx#_ENREF_105), [106](file:///C:\Users\Jude\AppData\Local\Temp\PSCI-D-16-00055R1%20v6.docx#_ENREF_106)] |
| Proteasome subunit alpha type | Multicatalytic ATP-dependent proteinase that cleaves peptides with Arg, Phe, Tyr, Leu, and Glu adjacent to the leaving group at neutral or slightly basic pH. | [[23](file:///C:\Users\Jude\AppData\Local\Temp\PSCI-D-16-00055R1%20v6.docx#_ENREF_23)] |
|  | Has a role in oxidative stress in sheep. | [[107](file:///C:\Users\Jude\AppData\Local\Temp\PSCI-D-16-00055R1%20v6.docx#_ENREF_107)] |
| Proteasome subunit beta type | Multicatalytic trypsin-like proteinase that with a broad specificity. | [[23](file:///C:\Users\Jude\AppData\Local\Temp\PSCI-D-16-00055R1%20v6.docx#_ENREF_23)] |
|  | Plays a role in sheep naturally infected with scrapie. | [[108](file:///C:\Users\Jude\AppData\Local\Temp\PSCI-D-16-00055R1%20v6.docx#_ENREF_108)] |
| Protein-serine/threonine kinase | ATP binding, receptor signalling protein activity, receptor signalling, transforming growth factor beta binding and transforming growth factor beta receptor activity. | [[23](file:///C:\Users\Jude\AppData\Local\Temp\PSCI-D-16-00055R1%20v6.docx#_ENREF_23)] |
| Protein-tyrosine-phosphatase | Regulates growth and replacement of intestinal epithelial cells | [[109](file:///C:\Users\Jude\AppData\Local\Temp\PSCI-D-16-00055R1%20v6.docx#_ENREF_109)] |
|  | Plays a role in paratuberclosis infection in sheep. | [[110](file:///C:\Users\Jude\AppData\Local\Temp\PSCI-D-16-00055R1%20v6.docx#_ENREF_110)] |
| Prothrombin | blood coagulation | [[111-113](file:///C:\Users\Jude\AppData\Local\Temp\PSCI-D-16-00055R1%20v6.docx#_ENREF_111)] |
| Superoxide dismutase [Cu-Zn] | preventing cell destruction by scavenging on radicals | [[23](file:///C:\Users\Jude\AppData\Local\Temp\PSCI-D-16-00055R1%20v6.docx#_ENREF_23)] |
| Transaldolase | Balances metabolites in the pentose-phosphate pathway. | [[23](file:///C:\Users\Jude\AppData\Local\Temp\PSCI-D-16-00055R1%20v6.docx#_ENREF_23)] |
|  | Possibly contributes to the pathogenesis of encephalitis in sheep. | [[114](file:///C:\Users\Jude\AppData\Local\Temp\PSCI-D-16-00055R1%20v6.docx#_ENREF_114)] |
| Uricase | Catalyses the oxidation of uric acid to 5-hydroxyisourate for it its conversion into allantoin. | [[23](file:///C:\Users\Jude\AppData\Local\Temp\PSCI-D-16-00055R1%20v6.docx#_ENREF_23), [115](file:///C:\Users\Jude\AppData\Local\Temp\PSCI-D-16-00055R1%20v6.docx#_ENREF_115)]. |
|  |  |  |
